# Anomaly Detection and Remaining Useful Life Prediction for Turbofan Engines with a Key Point-Based Approach to Secure Health Management

**DOI:** 10.3390/s24248022

**Published:** 2024-12-16

**Authors:** Yuntao Duan, Tao Zhang, Dunhuang Shi

**Affiliations:** 1 School of Mechatronic Engineering, Xi’an Technology University, No.2 Xuefuzhonglu Road, Weiyang District, Xi’an 710021, China; duanyuntao@st.xatu.edu.cn (Y.D.); shidunhuang@st.xatu.edu.cn (D.S.); 2 School of Computer and Software, Nanyang Institute of Technology, No. 80 Changjiang Road, Nanyang 473004, China

**Keywords:** remaining useful life, prognostics and health management, key point, convolution

## Abstract

Aero-engines, particularly turbofan engines, are highly complex systems that play a critical role in the aviation industry. As core components of modern aircraft, they provide the thrust necessary for flight and are essential for safe and efficient operations. However, the complexity and interconnected nature of these engines also make them vulnerable to failures and, in the context of intelligent systems, potential cyber-attacks. Ensuring the secure and reliable operation of these engines is crucial as disruptions can have significant consequences, ranging from costly maintenance issues to catastrophic accidents. The innovation of this article lies in a proposed method for obtaining key points. The research method is based on convolution and the basic shape of convolution. Through feature fusion, a self-convolution operation, a half operation, and derivative operation on the original feature data of the engine, two key points of the engine in the entire lifecycle are obtained, and these key points are analyzed in detail. Finally, the key point-based acquisition method and statistical data analysis were applied to the engine’s health planning and lifespan prediction, and the results were validated on the test set. The results indicate that the key point-based method proposed in this paper has promising prospects.

## 1. Introduction

The integration of artificial intelligence (AI) into critical systems, such as aero-engines, has heightened the need for robust security and operational reliability. Aero-engines, particularly turbofan engines, are highly complex systems that serve a fundamental role in aviation [1]. As essential components of modern aircraft, they provide the thrust required for flight and are crucial to ensuring safe and efficient operations [2,3]. However, their complexity and interconnectedness also increase their vulnerability to failures and, within the context of intelligent systems, to potential cyber-attacks. Securing the reliable operation of these engines is therefore critical, as any disruption can lead to severe consequences, including costly maintenance or catastrophic failure. In the era of advanced intelligent systems, the early detection of anomalies and the accurate prediction of the remaining useful life (RUL) of key components are vital for effective maintenance and safety.

PHM technology (prognostics and health management) involves two major aspects: fault prediction and health management. Fault prediction, also known as the P (prognostics) part in PHM, mainly refers to diagnosing and predicting the current and future health status, performance degradation, and occurrence of faults based on system history and current monitoring data. Health management, also known as HM (health management) in PHM, mainly refers to the ability to make appropriate plans and decisions and to achieve coordination for tasks, maintenance, and support activities based on diagnostic, assessment, and predictive results combined with available maintenance resources and equipment usage requirements.

The prediction of the remaining useful life (RUL) of the transmitter has become an important task in PHM technology [4]. The purpose of predicting the remaining useful life is to predict the failure time of core components in advance, to reduce accident costs, and to avoid accidents [3]. The methods for predicting the remaining life of aircraft engines can be divided into three categories: methods based on physical degradation models, based on mathematical statistical models, and based on neural network models.

Methods based on physical degradation models mainly use physics knowledge to establish degradation models. For example, Hoeppner, D.W. et al. established a fatigue crack development model using knowledge of fracture mechanics and fatigue crack propagation laws in their paper [5], which was then used for RUL prediction. In [6], Le Son, K et al. applied the Hertz contact dynamics theory of ball bearing raceways to establish a life prediction model for bearings. Sanchez et al. proposed a method in [7] to develop a degradation model using physically informed learning to estimate the remaining lifespan of the system. This method involves combining physical knowledge about the system with a set of data obtained from similar systems that have failed in the past to establish a set of constraints on the evolution of the system’s health status over time. The physical knowledge involved includes a set of approximate system dynamics and aging differential equations. The prediction method based on physical models generally has good prediction performance [8]. However, due to the complex mechanical structure and uncertain application environment of the equipment, there is great uncertainty about the accuracy of the model [9,10].

Based on mathematical statistical modeling methods, degradation models are mainly established using methods such as the Wiener model, Kalman filter, Bayesian model, etc. For example, Liu, Sujuan et al. [11] addressed the phenomenon of the nonlinear degradation of engine performance parameters over time and modeled the degradation process of a single degradation signal using a nonlinear Wiener process. Li, N. et al. proposed the RUL prediction method (MSDFM) in [12]. In this method, the inherent degradation process of the system state is represented by a state transition function that follows the Wiener process. Liu, X. et al. constructed an underdetermined EKF estimator in [13] and applied it to the health state estimation of aircraft engines to solve the problem of limited available sensor data and unknown health parameters. GIA Quoc Bao Tran et al. proposed a mixed-class Kalman observer for general hybrid systems with linear (time-varying) dynamics and output mapping in [14], where the jump time of the solution is precisely known, and the exponential stability of the estimation error is demonstrated at any fast rate. Olís-Martín, D. et al.’s model selection in [15] was carried out through Bayesian optimization using repeated random down-sampling validation methods. In [16], Pin, Lim et al. proposed the switching Kalman filter (SKF) method, which can select the most likely degradation mode and make better predictions. In [17], Hu Y et al. quantified prediction uncertainty using improved Gaussian mixture distribution variational Bayesian inference (IVBI). The method proposed by Mosallam, A. et al. in [18] ultimately uses a discrete Bayesian filter to estimate the degradation state.

The prediction method based on neural networks has the advantages of high efficiency and low cost [19]. The main neural networks used include MLP, CNN, DCNN, LSTM, and various hybrid prediction methods. For example, in [20], F. Heimes first used an MLP architecture model, which features data from 21 sensors and three operating conditions, and won second place in the competition that year. Babu, G.S. et al. first used a CNN for RUL prediction in their paper [21]. Li, X. et al. proposed a data-driven method for prediction using deep convolutional neural networks (DCNNs) [22], which allows for normalized raw collected data to be directly used as input to the proposed network without requiring prior knowledge in prediction and signal processing. Chaoub, Alaaeddine. et al. used an end-to-end deep learning model based on multi-layer perceptron and long short-term memory layers (LSTM) to predict the RUL in [23]. After normalizing all data, the input is directly fed to the MLP layer for feature learning, and then it is fed to the LSTM layer to capture temporal dependencies, and finally fed to other MLP layers for RUL prediction. Rosero, Raúl et al. achieved accurate time series prediction in [24] by integrating function time series regression with joint learning (FL) by implementing a function multilayer perceptron (FMLP) while protecting data privacy. Sánchez Ruiz et al. combined a CNN with a GRU in [25] to obtain new features. The GRU collected temporal factors and then transformed them into one-dimensional vectors. Liu, Huixiang et al. combined CNN with LSTM and introduced shared encoding networks and domain adaptation mechanisms in their paper [26] to reduce data distribution differences between the source and target domains. Zhao C et al. proposed a dual-channel hybrid prediction model based on a CNN and bidirectional LSTM networks in their paper [27]; the model uses a CNN to extract features from time series data and considers the temporal sequence of the data using long short-term memory (LSTM) networks. Lee, Juseong et al. proposed a convolutional neural network with Monte Carlo dropout to estimate the distribution of the RUL [28].

During the experimental process, after performing some basic operations on the original feature data of the engine, it was found that there were two key points in the entire lifecycle of the engine. A detailed analysis of the key points revealed that all engines have key points, and the key point positions of different engines are not the same. Therefore, the analysis method based on engine key points can be applied to health planning and predicting the remaining life of turbofan engines. The analysis method based on engine key points can solve the specific requirements of complex mechanical structures and uncertain equipment application environments based on physical models and mathematical probability models. It can simplify calculations without relying on neural network training models. The effectiveness of this method was validated on the FD002 subset library of NASA’s C-MAPSS.

The rest of this article is structured as follows: The second section focuses on introducing the relevant work of this study. Section 3 discusses the methods and inferences for obtaining key points, while Section 4 analyzes the properties of key points. Section 5 presents applications based on key points. Finally, Section 6 presents the conclusions and future work.

## 2. Related Work

### 2.1. Convolution and Its Properties

Convolution is the sum of two variables multiplied within a certain range [29]. If the variables of the convolution are the correlation functions x(t) and h(t), then the convolution result is

(1) $$y(t)=x(t)*h(t)=\int_{-\infty }^{\infty }x(p)h(t-p)dp$$

Among them, p is the integral variable, t is the amount that causes h(−p) to shift, and * represents convolution.

If the convolutional variables are discrete sequences, x(n) and h(n), then the convolution result is

(2) $$y(n)=x(n)*h(n)=\sum_{i=-\infty }^{\infty }x(i)h(n-i)$$

Among these, * represents convolution; h(−i) is the result of taking the inverse of h(i); and the n of h(n−i) is the amount that causes the displacement of h(−i).

#### 2.1.1. One-Dimensional “Full” Convolution

**Figure 1:**
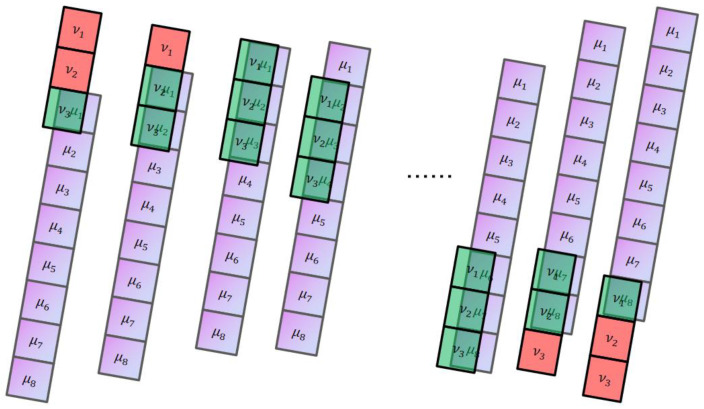
The calculation process of “full” convolution with two one-dimensional vectors.

Assuming μ and ν are one-dimensional vectors, the lengths of μ and ν are n and m, respectively. The full convolution calculation process of μ and ν:ν moves along the order of μ. For each position moved, the corresponding positions are multiplied and then added and summed. The length of the full convolution vector is n+m−1. The calculation process of one-dimensional full convolution is shown in Figure 1, [30].

#### 2.1.2. One-Dimensional “Same” Convolution

**Figure 2:**
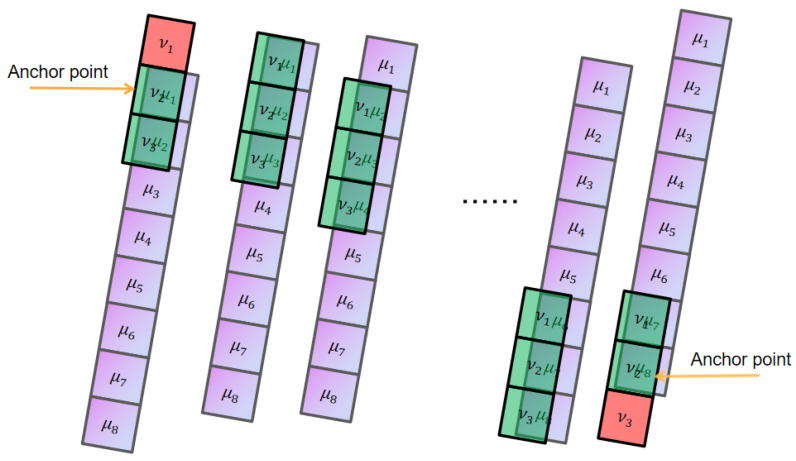
The calculation process of “same” convolution of two one-dimensional vectors.

The convolution kernel ν has an anchor point, which is moved to each position of μ in order of anchor position. The corresponding positions are multiplied, added, and summed to obtain the convolution result. After the same convolution process is carried out, the length remains n. The calculation process of one-dimensional same convolution is shown in Figure 2, [31].

The convolution of one-dimensional vectors satisfies the following properties (where f, g, g1, and g2 are all continuous functions or discrete sequences):

(3) Commutative law: f∗g=g∗f

(4) Associative law: (f∗g1)∗g2=f∗(g1∗g2)

(5) Distributive law: f∗(g1+g2)=f∗g1+f∗g2

(6) Linear properties: (αf)∗g=α(f∗g), α is constant

(7) $$\mathrm{Derivative}\;\mathrm{property}\text{:}\;\;\frac{d}{dt}(f*g)=\frac{d}{dt}(f)*g=f*\frac{d}{dt}(g)$$

(8) $$\mathrm{Time}\;\mathrm{invariance}\text{:}\;\mathrm{if}\;S_{\tau }(f)(t):=f(t-\tau ),\;\mathrm{then}\;(S_{\tau }(f))*g=S_{\tau }(f*g)$$

### 2.2. C-MAPSS Dataset

The C-MAPSS dataset [32] is a real simulation of engine degradation for large commercial turbofan aircraft provided by NASA, which records engine flight conditions and different failure modes. It includes four subsets (FD001, FD002, FD003, and FD004). Each subset contains 26 variables, of which 21 are sensor readings. The information of the C-MAPSS dataset is shown in Table 1, the collection indicators of 21 sensors are shown in Table 2, and the information of each column of the dataset is shown in Table 3.

Each sub dataset contains corresponding training and testing datasets. In the training dataset, the sensor monitoring data of each unit is complete, reflecting the entire lifecycle of the turbofan engine from initiation to failure. The testing dataset contains fragmented data of the same turbofan engine lifecycle. The dataset contains the monitoring data of turbofan engine performance components at different times, mainly including fan blades (fan), a low-pressure compressor (LPC), high-pressure compressor (HPC), combustion chamber (combustor), low-pressure rotor (N1), high-pressure rotor (N2), high-pressure turbine (HPT), low-pressure turbine (LPT), and nozzle. The data have temporal characteristics and reflect the degradation of the turbofan engine’s performance.

### 2.3. Custom Description

For the convenience of explanation, some operations and variables used in this article are defined as follows.

Original features: Each subset of the C-MAPSS database contains 26 variables, excluding the engine ID variable and the engine’s current lifecycle variable; the remaining 24 variables are defined as original features, specifically referring to the three operating conditions and 21 sensor data points collectively referred to as features. The variables formed after operating on these 24 original feature variables are also defined as features in this article.

Feature fusion: The feature fusion described in this article refers to the process of fusing 24 original feature variables into one-dimensional variables using the same convolution.

ff: The variable after feature fusion is called ff, corresponding to ${ff}_{train\_i}$ and ${ff}_{test\_i}$, where the subscripts train_i and test_i represent the i-th engine in the training set and the i-th engine in the testing set, respectively.

Self-convolution: The self-convolution defined in this article refers to the full convolution operation between the variable and itself.

fsf: The feature vector obtained from the self-convolution of feature variable ff is fsf, corresponding to ${fsf}_{train\_i}$ and ${fsf}_{test\_i}$, where the subscripts train_i and test_i represent the i-th engine in the training set and the i-th engine in the testing set, respectively.

fs: If the length of ff is l, then the length of feature fsf after the self-convolution operation is 2l−1. The first half of fsf, which is the length of l, it is taken and defined as variable fs, which includes ${fsf}_{train\_i}$ and ${fsf}_{test\_i}$, where the subscripts train_i and test_i represent the i-th engine in the training set and the i-th engine in the testing set, respectively.

Hp: The lifecycle value of feature variable A at its maximum inflection point value is Hp, which includes $Hp_{train\_i}$ and $Hp_{test\_i}$, where the subscripts train_i and test_i represent the i-th engine in the training set and the i-th engine in the testing set, respectively.

Calculate the derivative: A differentiation calculation is performed on one-dimensional discrete vector fs.

fss: Variable fss is obtained by performing a differentiation calculation on feature variable fs. It also includes ${fss}_{train\_i}$ and ${fss}_{test\_i}$, and the subscripts have the same meanings as stated before.

Lp: The lifecycle value of feature variable fss at its minimum inflection point value is Lp, which includes $Lp_{train\_i}$ and $Lp_{test\_i}$, and the subscripts have the same meanings as stated before.

## 3. Acquisition and Inference of Key Points

### 3.1. Acquisition of Key Points

The detailed description of the process for obtaining key points is as follows.

Firstly, feature fusion is performed on 24 original feature variables to obtain a one-dimensional feature variable, ff. Convolution fusion operations are performed on 24 original features in the “same” way, and finally, one-dimensional variables ff with unchanged lengths are obtained. The pseudocode for the convolution fusion process is shown in Table 4.

**Figure 3:**
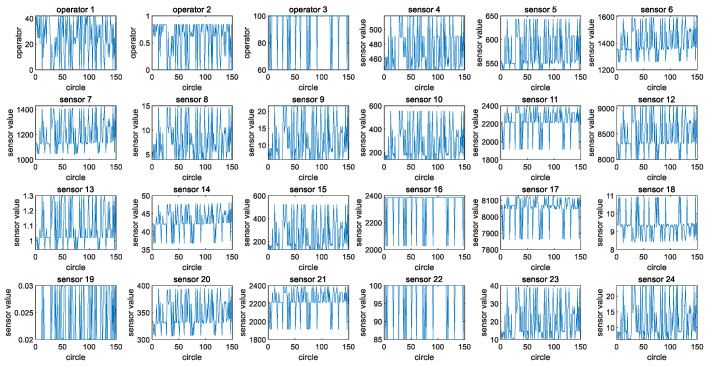
The 24 original feature maps of engine #1 in the training set (representing operating conditions 1–3 and 21 sensor data points, respectively).

**Figure 4:**
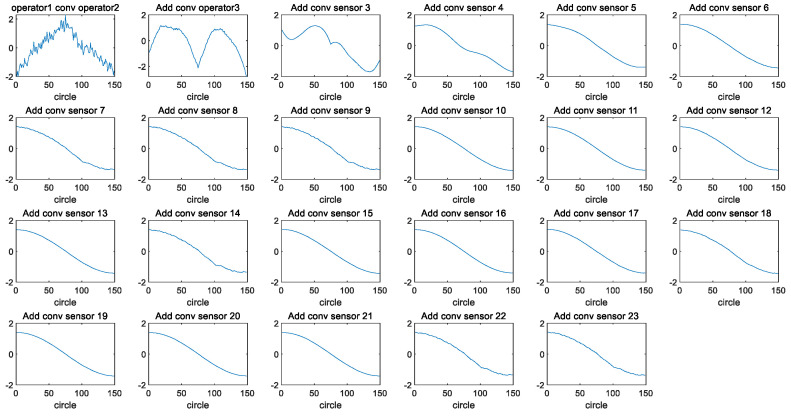
The feature fusion process of the 24 raw features of the training set for engine #1.

**Figure 5:**
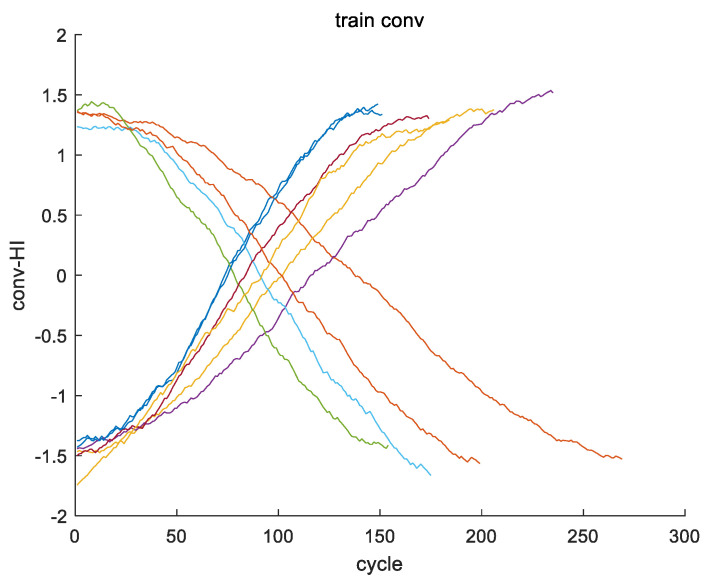
The images of variables ${ff}_{train\_1}\sim {ff}_{train\_10}$ after the feature fusion of engines #1~#10 in the training set.

The 24 original features of engine #1 in the training set of FD002 are taken as an example (operating conditions 1–3 and 21 sensor data points, respectively). Figure 3 shows the images of the 24 original features of the training set for engine #1. Figure 4 shows the fusion process of 24 original features in the training set for engine #1, resulting in the one-dimensional variable ${ff}_{train\_1}$. Figure 5 further illustrates the images of variables ${ff}_{train\_1}\sim {ff}_{train\_10}$ after the feature fusion of engines 1–10 in the training set.

According to the convolution property commutative law (Formula (3)) and associative law (Formula (4)), it can be seen that the convolution fusion order of the original features can be changed without affecting the fused result. According to the time invariant property of convolution (Formula (8)), it can be seen that the fused features will not change their correspondence with time.

**Figure 6:**
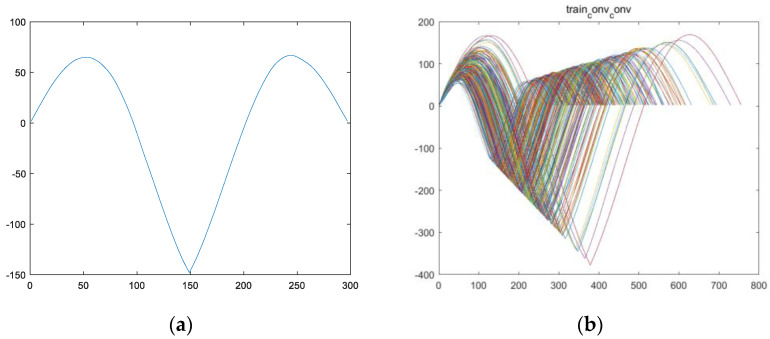
An image of vector fsf of the training set for the engine, which was obtained by performing a self-convolution operation on vector ff. (**a**) The ${fsf}_{train\_1}$ image of the training set for engine #1; (**b**) the fsf images of all engines in the training set (including ${fsf}_{train\_1}\sim {fsf}_{train\_260}$).

Secondly, the obtained convolutional fusion feature ff (assuming a length of l) is subjected to a self-convolution operation to obtain the feature vector fsf (with a length of 2l−1). For example, the fusion feature ${ff}_{train\_1}$ is obtained from the convolution fusion of engine #1 in the training set, and the feature vector ${fsf}_{train\_1}$ is obtained from the self-convolution operation of ${ff}_{train\_1}$. Figure 6a,b show the fsf images of all engines in the training set.

Once again, as feature variable fsf is obtained through a self-convolution operation, according to convolution Formula (2) and properties (3–8), it can be seen that variable fsf is symmetric and the image is symmetric. This can also be verified from Figure 6.

**Figure 7:**
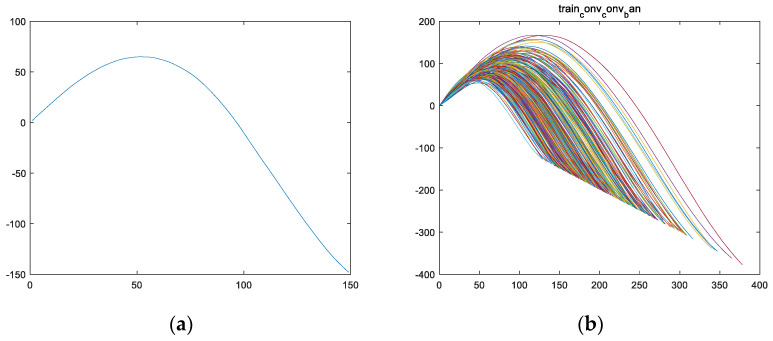
An image of vector fs of the training set for the engine, which was obtained by taking half of the operation for vector fsf. (**a**) The ${fs}_{train\_1}$ image of the training set for engine #1; (**b**) the fs images of all engines in the training set (including ${fs}_{train\_1}$~${fs}_{train\_260}$.

Half of feature variable fsf is used to obtain feature variable fs. Assuming the length of fsf is 2l−1, then the length of fs is l, as shown in Figure 7a for image ${fs}_{train\_1}$ and Figure 7b for images of all engines fs in the training set.

**Figure 8:**
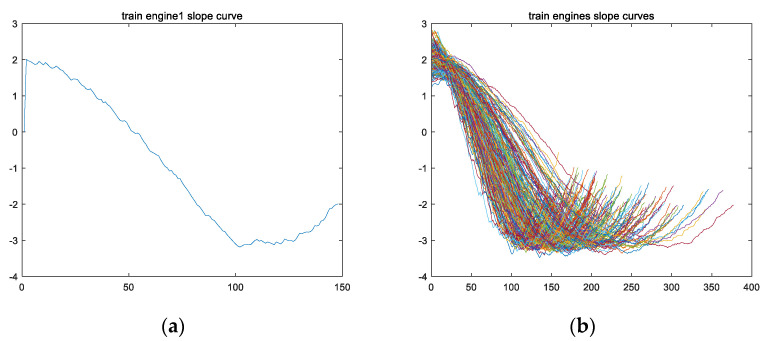
The image of vector fss of the engine training set, which was obtained by performing a derivative calculation operation on vector fsf. (**a**) The ${fss}_{train\_1}$ image of the training set for engine #1; (**b**) the fss images of all engines in the training set (including ${fss}_{train\_1}\sim {fss}_{train\_260}$).

Fourthly, a derivative calculation is performed on feature variable fs to obtain feature variable fss. Taking the training set for engine #1 as an example, the variable ${fss}_{train\_1}$ is obtained from the derivative calculation on feature variable ${fs}_{train\_1}$, and the curve of variable ${fss}_{train\_1}$ is shown in Figure 8a; Figure 8b shows the curves of variable fss for all engine training sets.

### 3.2. Inference

Based on the above operations in Section 3.1, we observed the following. 

**Figure 9:**
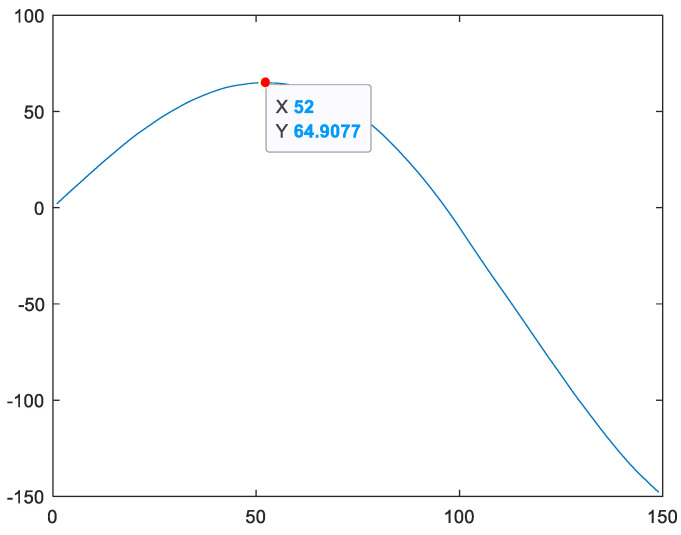
Taking variable ${fs}_{train\_1}$ of engine #1 in the training set as an example, the first key point $Hp_{train\_1}$ is the position of the maximum value of curve ${fs}_{train\_1}$.

Firstly, from Figure 7, it can be observed that the shape of feature vector fs presents a parabolic shape with the opening facing downwards. Therefore, vector fs has a maximum value, and the location of this maximum value is named the first key point Hp. Taking variable ${fs}_{train\_1}$ of the training set for engine #1 as an example, the highest point of the curve of variable ${fs}_{train\_1}$ (i.e., the maximum value of variable ${fs}_{train\_1}$) is the first key point $Hp_{train\_1}$, as shown in Figure 9.

**Figure 10:**
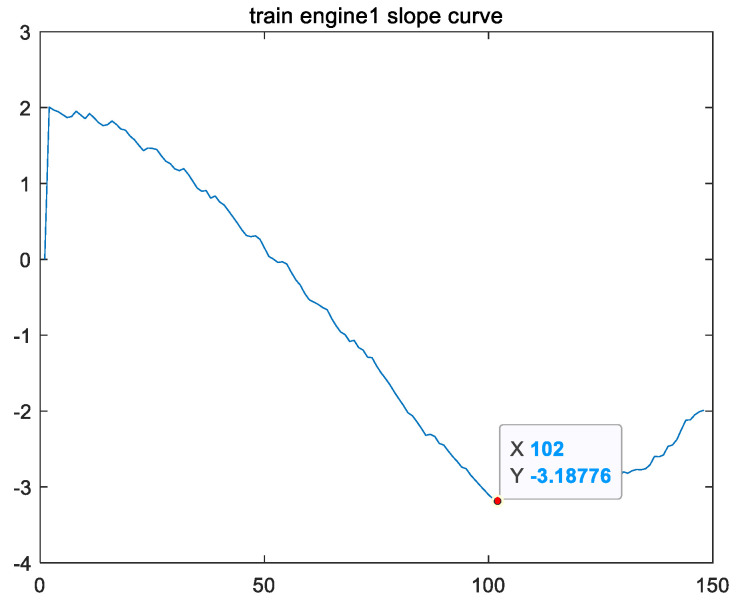
Taking variable ${fss}_{train\_1}$ from the training set for engine #1 as an example, the second key point $Lp_{train\_1}$ is the position of the minimum value of ${fss}_{train\_1}$’s curve.

Secondly, from Figure 8, it can be observed that the shape of feature vector fss presents a parabolic shape with an upward opening. Therefore, vector fss has a minimum value, and the location of this minimum value is named the second key point, Lp. Taking variable ${fss}_{train\_1}$ of the training set for engine #1 as an example, the curve of variable ${fss}_{train\_1}$ has the lowest point (i.e., the minimum value of variable ${fss}_{train\_1}$) as the second key point, $Lp_{train\_1}$, as shown in Figure 10.

**Figure 11:**
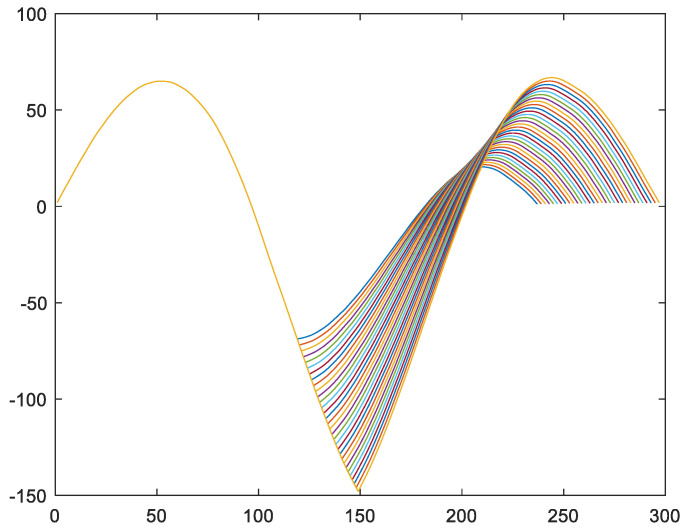
Taking the training set for engine #1 as an example, we extract vector ${ff}_{train\_1}$ by setting different truncation points to obtain different lengths of vector ${ff}_{train\_1}$ in order to verify whether the position of the first key point $Hp_{train\_1}$ of vector ${ff}_{train\_1}$ is the same under different length conditions.

We conducted experiments to test whether the position of key point Hp changes over time throughout the entire lifecycle of the engine. Taking the feature variable ${ff}_{train\_1}$ of engine #1 in the training set as an example, its lifecycle is 149. We start by truncating from 80% of the length to obtain feature variables with a length of 119, ..., 149. We perform self-convolution operations and plot curves to obtain the final image, as shown in Figure 11.

From the experimental results in Figure 11, it can be seen that the position of key point $Hp_{train\_1}$ remains unchanged as the time length of the extracted vector increases from 119 to 149.

After the appearance of key point Lp and before the end of its lifecycle, we also tested whether the position of point Lp would change with the increase in time period through experiments. Taking the feature variable ${fss}_{train\_1}$ of engine #1 in the training set as an example, its lifecycle is 149 and the key point ${Lp}_{train\_1}$ are located at 101. We start by truncating from 80% of the length to obtain feature variables with a length of 119, ..., 149, with variable lengths located after 101 and before 149. The final image obtained is the same as that shown in Figure 8, as the sequence curve of length 120 covers the curve of length 119, ..., and the curve with a length of 149 covers all previous curves, so it displays the same image as that shown in Figure 8. Therefore, for the entire lifecycle of the same engine, the position of key point Lp is unchanged.

From this, we can draw the following conclusions:

Conclusion 1: For the entire lifecycle of any engine, there must be two key points: Hp and Lp.

Conclusion 2: Throughout the entire lifecycle, the positions of key points Hp and Lp differ for different engines.

Conclusion 3: Throughout the entire lifecycle, the key points Hp and Lp of the same engine are fixed in position.

## 4. An Analysis of the Key Points

### 4.1. The First Key Point Analysis

**Figure 12:**
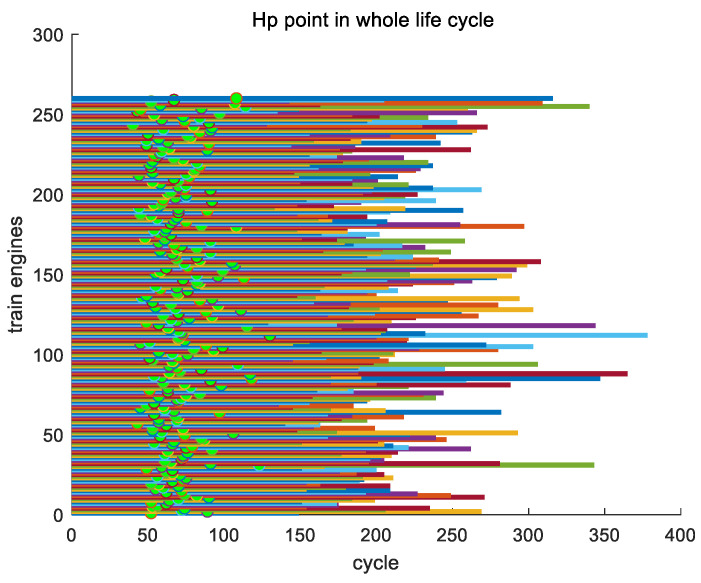
The position of the first key point Hp throughout its lifecycle.

From the inference mentioned earlier, there is a key point Hp in the entire lifecycle of each engine, and the timing of the appearance of key point Hp varies among different engines. Figure 12 shows the position of the first key point Hp of all engines in the training set throughout their entire lifecycles.

**Figure 13:**
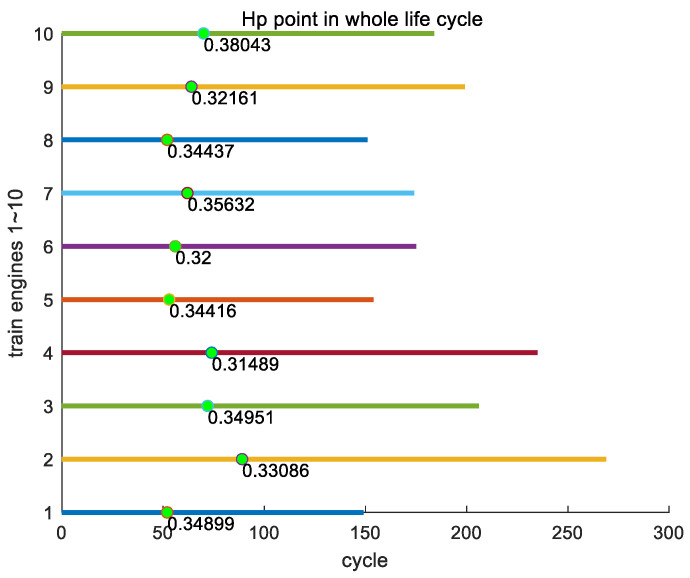
The proportions of key point Hp in the entire lifecycles of engines #1~#10 in the training set.

Here, we analyze the proportion of the occurrence time of key point Hp for all engines in the entire training set to their entire lifecycle. In the entire training set, the earliest appearance of key point Hp is in engine #136, with a position ratio of 0.2875 throughout its entire lifecycle; the latest appearance of key point Hp is in engine #148, which accounts for 0.40502 of its entire lifecycle; and the average proportion of the occurrence of key point Hp in its entire lifecycle is 0.3403. In Figure 13, as an example, we show the proportions of key point Hp in the entire lifecycles of engines #1~#10 in the training set.

### 4.2. The Second Key Point Analysis

**Figure 14:**
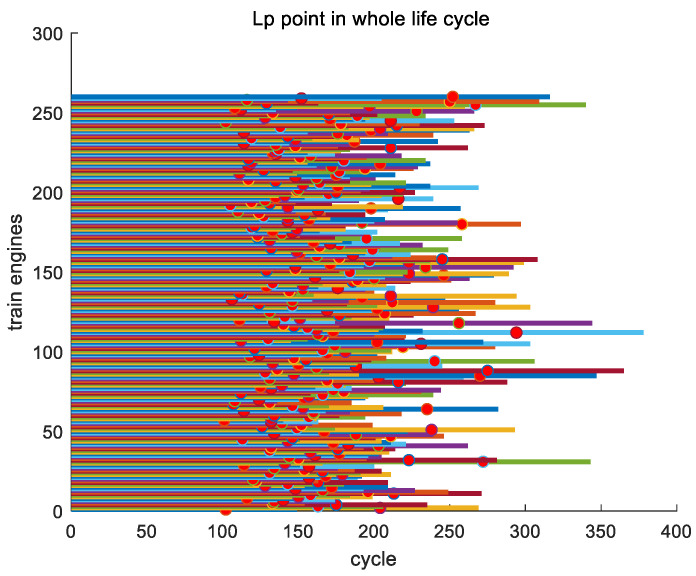
The positions of the second key point Lp for all engines in the training set throughout its entire lifecycle.

From the inference mentioned earlier, there is a key point Lp in the entire lifecycle of each engine, and the timing of the appearance of key point Lp varies among different engines. Figure 14 shows the positions of the second key point Lp for all engines in the training set throughout their entire lifecycles.

**Figure 15:**
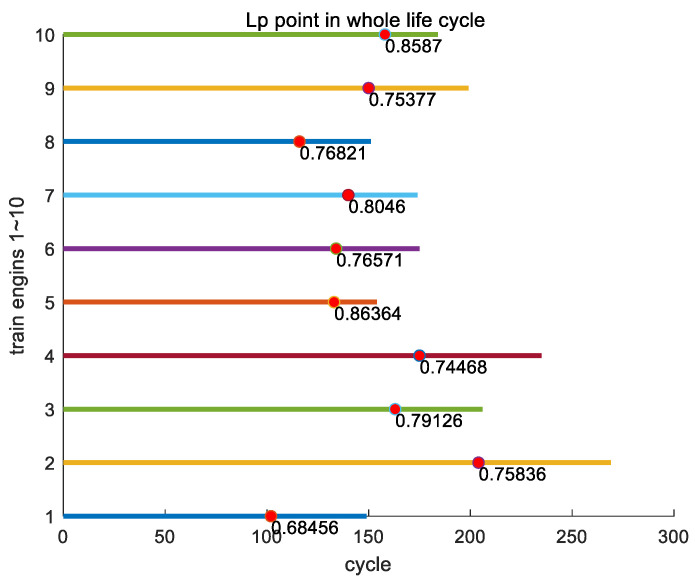
The proportions of key point Lp in the entire lifecycles of engines #1~#10 in the training set.

In this section, we analyze the proportion of key point Lp for all engines in the entire training set to their entire lifecycle. In the entire training set, the earliest appearance of key point Lp is engine #247, with a position ratio of 0.64975 throughout its entire lifecycle; the latest appearance of Lp is engine #196, which accounts for 0.90377 of its entire lifecycle; and the average proportion of the occurrence of Lp in its entire lifecycle is 0.77805. In Figure 15, as an example, we show the proportions of key point Lp in the entire lifecycles of engines #1~#10 in the training set.

### 4.3. Time Interval Analysis of Key Points Hp to Lp

**Figure 16:**
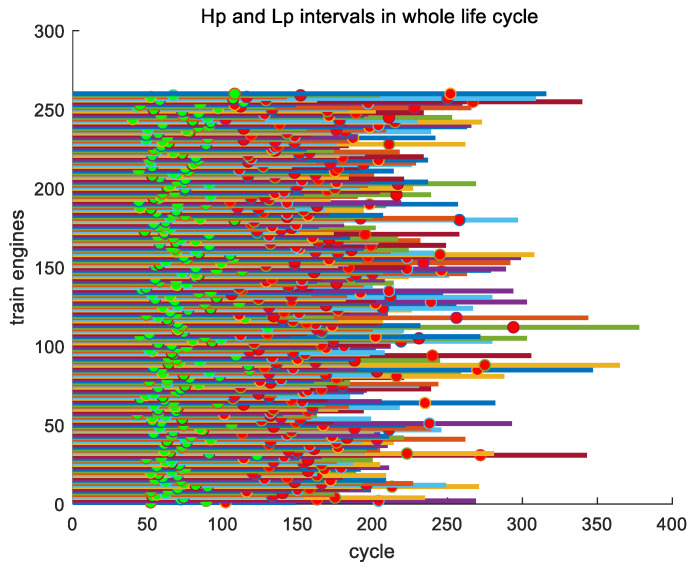
Positions of 2 key points, Hp and Lp, for all engines throughout their entire lifecycles in the training set.

The time interval between Hp and Lp refers to the time elapsed from the first key point Hp to the second key point Lp. Figure 16 displays two key points, Hp and Lp, simultaneously throughout the entire lifecycles of all engines in the training set, with the distance between key points Hp and Lp being the time interval between them.

**Figure 17:**
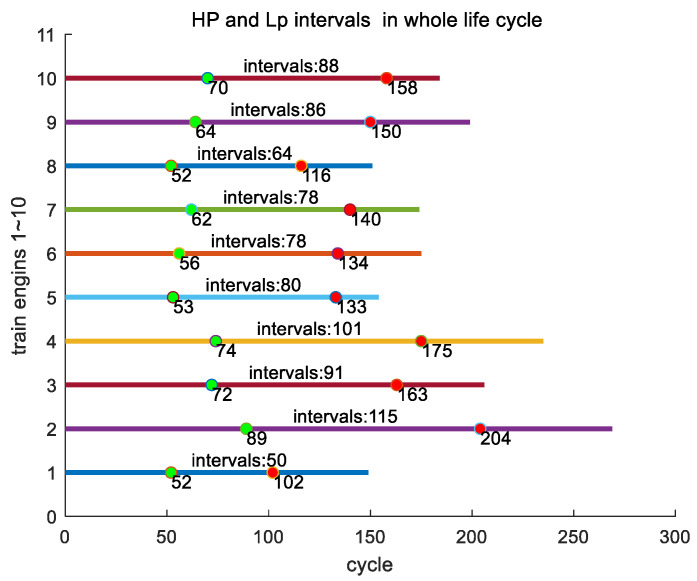
The time intervals of engines #1~#10 in the entire lifecycle of the training set.

We conducted an analysis of the proportion of this time interval throughout the entire lifecycle of its engine. In the entire training set, engine #1 has the smallest time interval between Hp and Lp, with a time interval of 50. The engine with the largest time interval is 112 with a time interval of 164. The average time interval between Hp and Lp is 90.4885. According to the proportion of time intervals in the lifecycle, engine #179 has the smallest proportion, accounting for 0.32597. The engine with the highest proportion is engine #55 with a maximum proportion of 0.54088. The average proportion of time intervals is 0.43775. As an example, in Figure 17, we show the time intervals of the training set for engines #1~#10 over their entire lifecycles.

### 4.4. Time Interval Analysis of Lp to End

In Section 4.2, the second key point, Lp, was analyzed, mainly focusing on the proportion of key point Lp in its engine lifecycle. In contrast, this section analyzes the time interval from key point Lp to the end of its lifecycle and its proportion throughout its lifecycle.

In the entire training set, the time interval from the Lp point to the end of the lifecycle accounts for the largest proportion of the entire lifecycle, with engine #247 accounting for 0.35025 or 35.025%. The engine with the smallest proportion is engine #196, which accounts for 0.09623 or 9.623%. The time interval from key point Lp to the end of the lifecycle accounts for an average of 0.222, or 22.2%, of the entire lifecycle.

### 4.5. Three Stages

The two key positions, Hp and Lp, divide the entire lifecycle into three stages, the [0,Hp] stage, [Hp,Lp] stage, and [Lp,end] stage, where end refers to the end of life.

[0,Hp] stage: The average proportion of position Hp in the entire lifecycle is 0.3403, accounting for 34% of the entire lifecycle, and the maximum proportion does not exceed 40.502%. It is in the early stage of equipment operation, and the equipment is very robust.

[Hp,Lp] stage: The average proportion of this stage in the entire lifecycle is 0.43775, which is approximately 43.8%, making it the largest proportion in the entire lifecycle. During this stage, the equipment operates relatively smoothly and degrades in a relatively stable manner.

[Lp,end] stage: The average proportion of the time interval from the critical point Lp to the end of its lifespan to the entire lifecycle of its engine is 22.2%, with a minimum proportion of 9.623%. The key position Lp indicates that this device has entered a rapid degradation stage, and its operation is unstable, which may terminate its lifespan at any time.

## 5. Application Based on Key Points

The two key points Hp and Lp proposed in this article have certain research and application value in the PHM (Prognostics Health Management) of core equipment and the prediction of the RUL (remaining useful life).

### 5.1. PHM Application

Prognostics Health Management (PHM), proposed to meet the requirements of autonomous support and diagnosis, is an upgraded development of condition-based maintenance (CBM) [33]. It emphasizes state perception in asset equipment management, monitoring equipment health status, frequent fault areas and cycles, and predicting the occurrence of faults through data monitoring and analysis, thereby significantly improving operational efficiency.

This article proposes a method that can easily determine key points Hp and Lp, which divide the entire engine lifecycle into three stages, as described in Section 4.5. In the application of PHM in turbofan engines, a management plan for PHM can be developed using the three stages described in Section 4.5. For example, when Hp has not yet appeared, it indicates that the equipment is in the early stage of operation, and the overall operation of the equipment is healthy. Maintenance work can be performed through simple routine inspections and maintenance. When Hp is detected, it indicates that the device has entered the second stage and can also roughly estimate the overall lifespan of the system. At this stage, a detailed equipment maintenance plan should be developed, and daily equipment inspections and maintenance should be carried out. When Lp is detected, it indicates that the equipment has entered the third stage, which accounts for 10% to 30% of the entire equipment lifespan. It is necessary to further strengthen equipment detection, prepare emergency plans, and be prepared for unexpected situations that may occur at any time.

### 5.2. RUL Prediction

#### 5.2.1. Evaluating Indicator

This article is based on the FD002 test set, which has multiple operating conditions and more difficulty in predicting the RUL compared to others. To evaluate performance, two main indicators are used: the Root Mean Square Error (RMSE) and the score for evaluating the RUL (introduced in [22,32,34,35,36]). Adaptive scores are introduced to penalize later predictions more severely than early predictions, as the latter are preferred.

These measures are defined by Equations (9) and (10), where ${rul}_{t}$ is the true RUL of period *t* and ${\hat{rul}}_{t}$ is the estimated RUL.

(9) $$RMSE=\sqrt{\frac{1}{N}\sum_{i=1}^{N}{\left({rul}_{t}-{\hat{rul}}_{t}\right)}^{2}}$$

(10) $$score=\left\{\begin{array}{c} \sum_{i=1}^{N}exp-\frac{{\hat{rul}}_{t}-{rul}_{t}}{13}-1\;\;\;\;\;\;\;\;\;{\hat{rul}}_{t}-{rul}_{t}<0\\ \sum_{i=1}^{N}exp\frac{{\hat{rul}}_{t}-{rul}_{t}}{10}-1\;\;\;\;\;\;\;\;\;\;\;\;\;\;\;{\hat{rul}}_{t}-{rul}_{t}\;\geq \;0 \end{array}\right.$$

#### 5.2.2. Obtaining Key Points of the Test Set

The key points of the engine test set are calculated using the method described in Section 3.

**Figure 18:**
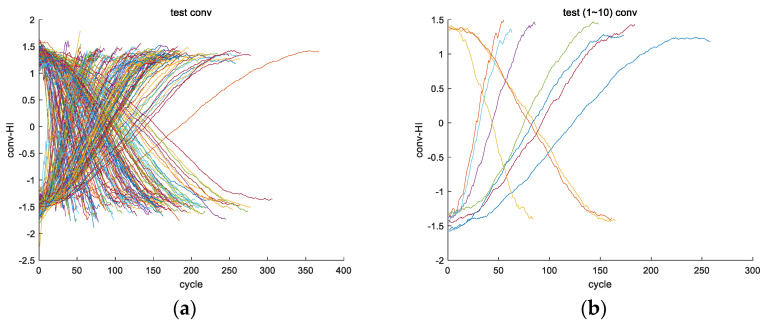
The image of variable ff generated by the test set for the engine after the feature fusion of the original features. (**a**) Variable ff is obtained after the fusion of all engines in the test set, which includes ${ff}_{test\_1}\sim {ff}_{test\_259}$. (**b**) The first 10 ff variables are obtained, i.e., ${ff}_{test\_1}\sim {ff}_{test\_10}$.

First, feature fusion is performed on the 24 original feature variables of each engine in the test set, and finally, a one-dimensional feature vector, ff, is obtained, as shown in Figure 18.

**Figure 19:**
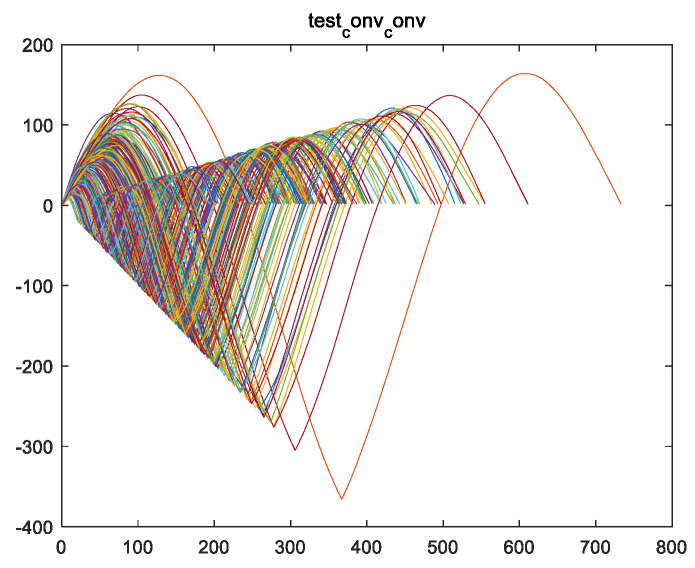
Images of vector fsf (i.e., ${fsf}_{test\_1}\sim {fsf}_{test\_259}$ images) for all engine test sets, where fsf is generated from vector ff through a self-convolution operation.

**Figure 20:**
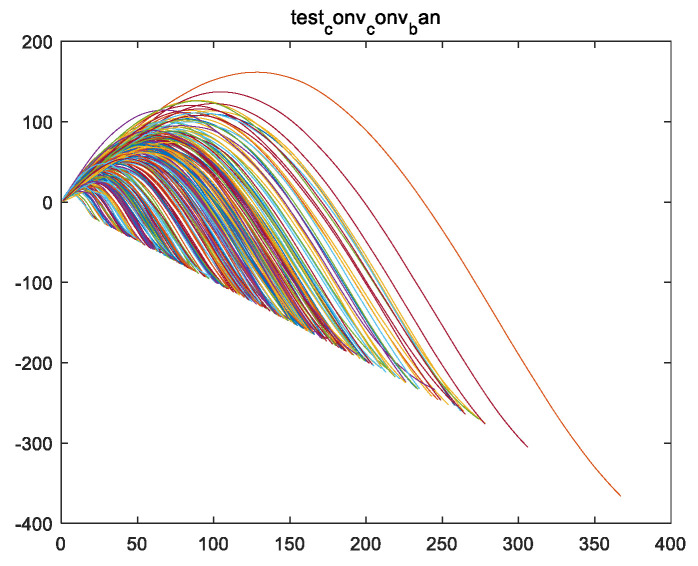
Vector fs images (i.e., ${fs}_{test\_1}\sim {fs}_{test\_259}$ images) of all engine test sets, where fs is generated from vector fsf by taking half of the operation.

After performing a self-convolution operation on variable ff using the test set, the feature vector fsf of the test set is obtained (as shown in Figure 19), and the feature variable fs is obtained (as shown in Figure 20).

From Figure 20, it can be seen that the curves of vector fs for all engine test sets are parabolic in shape, and all pass through the maximum point of the parabola (the maximum point represents the first key point Hp described in this article), indicating that all engine test sets are in the second and third stages (divided according to the three stages described in Section 4.5).

**Figure 21:**
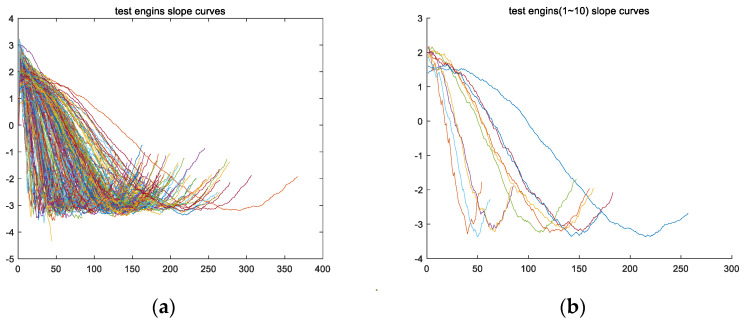
Vector fss images of engine test sets, where fss is calculated by taking the derivative of vector fs. (**a**) The image of vector fss for the entire test set (i.e., ${fss}_{test\_1}\sim {fss}_{test\_259}$). (**b**) Vector fss of the first 10 engines in the test set (i.e., ${fss}_{test\_1}\sim {fss}_{test\_10}$).

Fourthly, a derivative calculation is performed on feature variable fs to obtain feature variable fss. Figure 21 shows the vector fss images of the engine test sets.

**Figure 22:**
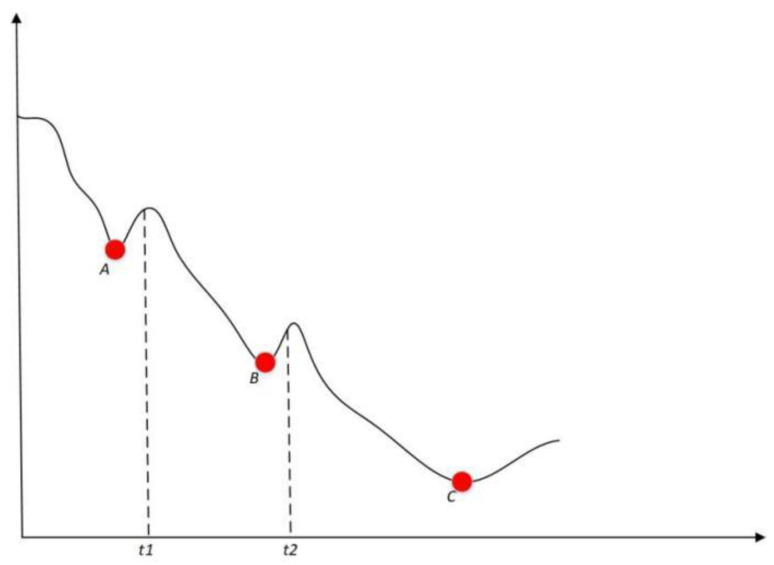
A schematic illustrating the reason why variable fss in the test set was mistakenly identified as the second key point Lp at the calculated minimum value.

Due to the fact that the engine test set has not yet reached the end of its lifespan, test set variable fss does not have the length of its entire lifecycle. Therefore, when calculating the second key point of test set variable Lp, the minimum value calculated may not necessarily be the minimum value of the entire lifecycle, and it may mistake the minimum value calculated at the current length for the second key point, Lp. As shown in Figure 22, the curve in the diagram represents the variable fss curve of a certain test engine. As shown in Figure 22, the curve in the schematic diagram represents the full lifecycle curve of variable fss of a certain engine. From the perspective of the full lifecycle, the true position of the second key point, Lp, of the engine is point C. But if the test length is [0,t1], the calculated position of the second key point Lp is point A. Similarly, if the test length is [0,t2], the calculated position of the second key point Lp is point B; in this case, points A and B are not the true key points.

How can we determine whether the minimum value calculated for variable fss in the current test set is the true second key point Lp? This can be determined based on the statistical analysis results of the “Time interval analysis of Lp to end” in Section 4.4 above.

In the entire training set, the engine with the smallest time interval from Lp to the endpoint is engine #196, accounting for 9.623%. The engine with the maximum time interval from Lp to the finish line is engine #247, accounting for 35.025%. A threshold is set here to calculate the time interval from point Lp to the endpoint of the test set. If the time interval is shorter than the set threshold, it is considered that the engine has not yet reached the true Lp position, and it is considered that the engine is running in the second stage. This type of engine is divided into ensemble TEST2. When the time interval is greater than the set threshold, it is considered that the Lp calculated by the engine is true, that is, it is considered that the engine is running in the third stage, and this type of engine is divided into the TEST1 set.

For example, when the time interval threshold from test set Lp to the endpoint is set to 30, it is determined that test set TEST1 has 110 engines. Test set TEST2 has 149 engines. When the time interval threshold from test set Lp to the endpoint is set to 18, it is determined that there are 187 engines in test set TEST1. Test set TEST2 has 72 engines.

Therefore, in the test set, a threshold is set to filter and distinguish between test engines that have and have not yet reached the third stage. Furthermore, the engine test set is divided into two parts: one part is the set of engine test sets that have reached the third stage and can obtain Hp and Lp (named TEST1); the other part is the engine test set (TEST2) that has not yet reached the third stage.

#### 5.2.3. Using the Method in Conjunction with Other Prediction Algorithms

As mentioned earlier, using the method of determining the positions of Hp and Lp, the original test set TEST is divided into two datasets: TEST1 (which includes the test engines at key positions Hp and Lp) and TEST2 (which only includes the test engines at position Hp). TEST2 only contains the location information of Hp, which has low prediction accuracy and is suitable as a rough estimate. The TEST1 set includes location information for two key points, Hp and Lp, with higher prediction accuracy and more practical significance. Therefore, in the actual RUL prediction process, the first step is to partition TEST1 and then use various current RUL prediction methods to make RUL predictions, resulting in significantly improved prediction results. Table 2 shows several mainstream methods used in this article, namely MLP, CNN, DCNN, and LSTM. The RUL evaluation results between the TEST set and the TEST1 set are compared.

According to Table 5, the prediction accuracy of the TEST1 set has significantly improved, with the score improving by about four times compared to the original TEST set prediction.

**Figure 23:**
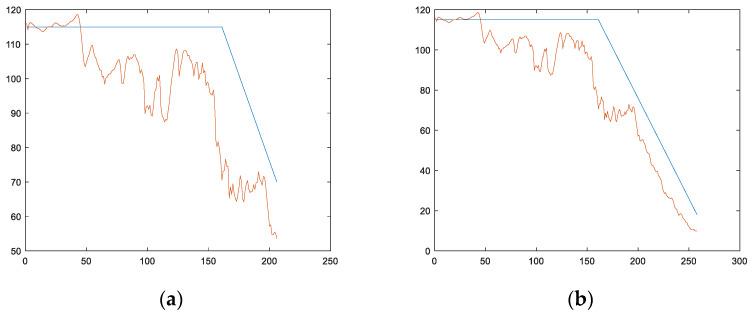
The test set for engine #1 is used as an example to compare the RMSE and score for two scenarios: when the length of variable ${fss}_{test\_1}$ is shorter than the true second key point ${LP}_{test\_1}$ and when the length of variable ${fss}_{test\_1}$ is greater than the true second key point ${LP}_{test\_1}$. (**a**) The predicted result when selecting variable ${fss}_{test\_1}$ with a length of 206 (the length is shorter than the actual second key point position). (**b**) The predicted result when the length of variable ${fss}_{test\_1}$ is 258 (longer than the actual second key point position).

Taking a single engine test set as an example, the length of the test set for engine #1 is 258, and the key points Hp and Lp calculated using the method in this article are 99 and 217, respectively. In order to compare the evaluation results of the length of the engine test set variable fss before and after the real second key point Lp, we first take 80% of the length of engine #1, which is 258 × 0.8 = 206, and this length is shorter than the Lp position. At this point, the evaluation result obtained is RMSE = 16.385689 with a score of 2.526965, and the image is shown in Figure 23a. When the total length is 258 and the length is greater than the Lp position, the evaluation result obtained is RMSE = 8.089673 with a score of 0.863176, as shown in Figure 23b.

#### 5.2.4. Using the Key Point Method Alone to Predict the RUL

In addition to being used in conjunction with other methods, as described in Section 5.2.3, the two key positions Hp and Lp can also be used separately to predict the engine’s RUL. This article provides two examples of usage.

Case 1: Two-point method

The two points mentioned here refer to key points Hp and Lp, which can form a two-dimensional coordinate point. The $Hp_{train\_i}$ and $Lp_{train\_i}$ values of the engine training set can form a two-dimensional coordinate point $\left(Hp_{train\_i},Lp_{train\_i}\right)$. Similarly, in the TEST1 engine, $Hp_{test\_i}$ and $Lp_{test\_i}$ form coordinates $\left(Hp_{test\_i},Lp_{test\_i}\right)$. The distance between two-dimensional coordinate points is utilized to determine the similarity between two points.

The method for predicting two points based on similarity involves the following steps:

First, take engine test set i and calculate its $Hp_{test\_i}$ and $Lp_{test\_i}$ values to form coordinates $\left(Hp_{test\_i},Lp_{test\_i}\right)$.

Second, calculate the distance between coordinates $\left(Hp_{test\_i},Lp_{test\_i}\right)$ and all training set coordinates $\left(Hp_{train\_j},Lp_{train\_j}\right)$.

Third, choose the engine training set train_j with the smallest distance as the most similar engine to engine test set test_i, that is, use the entire lifecycle of engine training set train_j as the entire lifecycle of engine test set test_i.

Fourth, calculate the RUL of the engine test set test_i and evaluate the RMSE and score.

For example, if the value of the coordinates $\left(Hp_{test\_1},\;Lp_{test\_1}\right)$ of engine 1 in the test set is (99,217), the distance from (99,217) to all training sets $\left(Hp_{train\_j},Lp_{train\_j}\right)$ is calculated, and then it is determined that the nearest training set coordinate is (98,216) with a distance of 1.0108, which corresponds to engine 81 in the training set. Using the lifecycle of engine 81 in the training set as the lifecycle of engine 1 in the testing set, the calculated RUL is 288 − 258 = 30. The RMSE is 12 and the score is 2.3201.

As another example, if the value of the coordinates $\left(Hp_{test\_65},Lp_{test\_65}\right)$ of engine #65 in the test set is (129,289), the distance from (129,289) to all training sets $\left(Hp_{train\_j},Lp_{train\_j}\right)$ is calculated, and then it is determined that the nearest training set coordinate is (49,108) with a distance of 2.1007, which corresponds to engine 69 in the training set. Using the lifecycle of engine 69 in the training set as the lifecycle of engine #65 in the testing set, the calculated RUL is 136 − 367 = −231, and the true RUL is 11. The RMSE is 242, and the score is 1.2150e + 08.

Case 2: Three-point method

Similarly to Case 1′s two-point method, in addition to key points Hp and Lp, the data $ratio\;of\;\frac{Hp}{Lp-Hp}$ is added to form the coordinates of three points (Hp,Lp,ratio).

$Hp_{train\_j}$, $Lp_{train\_j}$, and ${ratio}_{train\_j}$ of the engine training set can form a three-dimensional coordinate point $\left(Hp_{train\_j},\;Lp_{train\_j},\;{ratio}_{train\_j}\right)$. Similarly, in the TEST1 engine, $Hp_{test\_i}$, $Lp_{test\_i}$, and ${ratio}_{test\_i}$ form coordinates $\left(Hp_{test\_i},\;Lp_{test\_i},\;{ratio}_{test\_i}\right)$. The distance between three-dimensional coordinate points is utilized to determine the similarity between two points.

The steps of the three-point method are similar to the four steps used in the two-point method.

For example, if the value of the coordinates $\left(Hp_{test\_1},\;Lp_{test\_1},\;{ratio}_{test\_1}\right)$ of engine #1 in the test set is (99,217,0.8390), the distance from (99,217,0.8390) to all training sets $\left(Hp_{train\_j},Lp_{train\_j},{ratio}_{train\_j}\right)$ is calculated, and it is found that the nearest training set coordinate is (98,216,0.8305) with a distance of 1.4142, which corresponds to engine #81 in the training set. Using the lifecycle of engine #81 in the training set as the lifecycle of engine #1 in the testing set, the calculated RUL is 288 − 258 = 30. The RMSE is 12, and the score is 2.3201.

As another example, if the value of the coordinates $\left(Hp_{test\_65},Lp_{test\_65},{ratio}_{test\_65}\right)$ of engine #65 in the test set is (129,289,0.8063), the distance from (129,289,0.8063) to all training sets $\left(Hp_{train\_j},Lp_{train\_j},{ratio}_{train\_j}\right)$ is calculated, and it is found that the nearest training set coordinate is (130,294,0.7927) with a distance of 5.0990, which corresponds to engine #112 in the training set. Using the lifecycle of engine #112 in the training set as the lifecycle of engine #65 in the testing set, the calculated RUL is 378 − 367 = 11, and the true RUL is 11. The RMSE is 0, and the score is 0.

## 6. Conclusions

After analyzing the raw characteristic data of the entire cycle of turbofan engines, this article proposes a simple and effective method for obtaining key points. The main operational steps include feature fusion, a self-convolution operation, taking half of the operation, and a derivative operation. The key point-based method can be applied to health planning and predicting the remaining life of turbofan engines. The effectiveness of this method was validated on the FD002 subset library of NASA’s C-MAPSS. In the application of health planning, the key point method presented in this article can divide the lifecycle of the engine into three stages, which can help carry out rough health planning and predictions for the engine. In the application of remaining life prediction, other mainstream methods can be combined to improve prediction accuracy (the combination of methods such as MLP, CNN, DCNN, LSTM, etc., can improve prediction accuracy by four times compared to before), and they can also be used separately for prediction (such as the two-point method to obtain an RMSE of 12 and a score of 2.3201 in the test set for engine #1 and the three-point method to obtain an RMSE of 0 and a score of 0 in the test set for engine #65, achieving accurate prediction in some individual engines). In this era of advanced intelligent systems, the ability to detect anomalies early and predict the remaining useful life (RUL) of key components is not only vital for maintenance and safety but also for safeguarding against potential security threats that could compromise the integrity of the system. The key point acquisition method proposed in this article has the advantages of simplicity and practicality, and it can be quickly applied to health planning and predicting the remaining life of turbofan engines. The limitations of this study are as follows: The position of the second key point of the tested engine is easily misjudged. When using the key point method in combination with other mainstream methods, the improvement in testing accuracy is only significant after the second key point of the testing engine appears. When using the key point method alone for prediction, such as the two-point method, three-point method, etc., it essentially acts as a big data matching method, and the measurement accuracy should be based on the completeness of the large database.

Although the key point-based method proposed in this article has achieved certain experimental results, there is still much work to be conducted in the future. Firstly, in terms of applications based on key points, the application presented in this article serves as a starting point for further exploration, and there are still many applications that utilize key points that need to be further explored. Secondly, the two-point and three-point methods cited in this article have achieved very good results in some engine test sets but not in others. The analysis suggests that reasons may be due to noise in the original features and incomplete training set data. Further experimental verification is needed in the next step. Thirdly, most of the current RUL objective functions use piecewise functions, with fixed threshold values ranging from 110 to 150. The first key point position in this article is different for different engines and is a dynamic piecewise function threshold. The next step is to use the first key point proposed in this article as the dynamic threshold value for the piecewise function.

## Figures and Tables

**Table 1:** The composition of the C-MAPSS dataset.

Dataset	FD001	FD002	FD003	FD004
Training engines	100	260	100	249
Testing engines	100	259	100	248
Operating conditions	1	6	1	6
Fault modes	1	1	2	2
Number of training samples	20,632	53,760	24,721	61,250
Number of testing samples	13,097	33,992	16,597	41,215

**Table 2:** Data description of turbofan engine sensors.

Sensor No	Sensor Description	Units
1	Total temperature at fan inlet	°R
2	Total temperature at LPC outlet	°R
3	Total temperature at HPT outlet	°R
4	Total temperature at LPT outlet	°R
5	Pressure at fan inlet	psia
6	Total pressure in bypass duct	psia
7	Total pressure at HPC outlet	psia
8	Physical fan speed	rpm
9	Physical core speed	rpm
10	Engine pressure ratio	-
11	Static pressure at HPC outlet	rpm
12	Ratio of fuel flow to Ps30	pps/psi
13	Corrected fan speed	rpm
14	Corrected core speed	rpm
15	Bypass ratio	-
16	Burner fuel/air ratio	-
17	Bleed enthalpy	-
18	Demanded fan speed	rpm
19	Demanded corrected fan speed	rpm
20	HPT coolant bleed	lbm/s
21	LPT coolant bleed	lbm/s

**Table 3:** The parameter names of each column in the dataset.

Columns	1	2	3–5	6–26
Parameter Name	Engine id	Current lifecycle of engine	Operating conditions	Sensor data

**Table 4:** Convolutional fusion process.

Convolutional Fusion Process	
Input: train_cell{}, include all train engine data, every engine data size is *features × length* **Output: conv result, in which every engine data size is 1 × length**	
1: 2: 3: 4: 5: 6: 7: 8: 9: 10: 11:	for i = 1: numer of engines in the train sets Get the *i-th* engine data of the train_cell; the i-th engine data size is *features × length*; Get the i-th engine data’s first feature, the size is *1 × length*, named h1; for j = 2: *features* Get the ith engine data’s j-th *feature*, and convolution with *h1*, then reassign the result to *h1*, Central part of the convolution of the same size as *h1*,that is the h1’s size is *1 × length*. end the *h1* is the *i-th* engine convolution result and is saved in variable train_conv_cell. end

**Table 5:** Comparison of TEST and TEST1.

		MLP [34]	CNN [35]	DCNN [36]	LSTM [22]
TEST	RMSE	28.78	21.16	22.36	24.49
	Score	14,026.72	3815.85	10,412	4450
TEST1	RMSE	25.32	18.41	19.34	21.41
	Score	3543.43	1208.34	2467.62	1097

## Data Availability

The reach dataset is a public dataset, it can be downloaded form: https://data.nasa.gov/dataset/C-MAPSS-Aircraft-Engine-Simulator-Data/xaut-bemq (accessed on 5 December 2024).

## Abbreviation

Explanations of all the abbreviations used in this article.

RUL	Remaining Useful Life
AI	Artificial Intelligence
PHM	Prognostics and Health Management
MSDFM	Multi-Sensor Data Fusion Model
EKF	Extended Kalman Filter
SKF	Switching Kalman Filter
IVBI	Iterative Variational Bayesian Inference
MLP	Multilayer Perceptron
CNN	Convolutional Neural Networks
DCNN	Deep Convolutional Neural Network
LSTM	Long Short-Term Memory
FMLP	Functional Multilayer Perceptron
GRU	Gate Recurrent Unit
CBM	Condition-Based Maintenance
RMSE	Root Mean Square Error
